# Penguin Chicks Benefit from Elevated Yolk Androgen Levels under Sibling Competition

**DOI:** 10.1371/journal.pone.0042174

**Published:** 2012-07-30

**Authors:** Maud Poisbleau, Wendt Müller, David Carslake, Laurent Demongin, Ton G. G. Groothuis, Jeff Van Camp, Marcel Eens

**Affiliations:** 1 Department of Biology - Ethology, University of Antwerp, Wilrijk, Belgium; 2 MRC Centre for Causal Analyses in Translational Epidemiology (CAiTE), School of Social and Community Medicine, University of Bristol, Bristol, United Kingdom; 3 Behavioural Biology, Institute of Behavioural Neuroscience, University of Groningen, Groningen, The Netherlands; Monash University, Australia

## Abstract

Crested penguins (genus *Eudyptes*) have a peculiar hatching pattern, with the first-laid egg (A-egg) hatching after the second-laid egg (B-egg) and chicks from A-eggs typically having a much lower survival probability. Maternal yolk androgens have been suggested to contribute to the competitive superiority of the B-chick in southern rockhopper penguins *Eudyptes chrysocome*, given their important role in mediating sibling competition in other species. We therefore increased the yolk androgen levels in freshly-laid eggs and examined the consequences for sibling competition - *via* effects on embryonic developmental times, chick growth and early survival. We placed one androgen-treated egg and one control egg into each foster nest, matching them for mass, laying date and laying order. The androgen treatment did not significantly affect embryonic developmental times or chick measurements at hatching. However, elevated yolk androgen levels benefitted chick growth in interaction with the number of siblings in a brood. Chicks from androgen-treated eggs had faster growth in the presence of a sibling than chicks from control eggs. Under these circumstances they also had a higher survival probability. Thus maternal androgens appear to reinforce the observed hatching pattern, facilitating brood reduction. This contrasts to most previous studies in other species where yolk androgens have been shown to compensate for the negative consequences of delayed hatching within the brood hierarchy.

## Introduction

In most bird species, parents initially produce more offspring than they are able to raise [1]–[4]. This overproduction commonly results in an intense competition between siblings that may ultimately lead to the elimination of part of the brood. Parents influence the level of sibling competition by producing offspring that differ in age, size or quality. Parents achieve this in particular by varying the onset of incubation before clutch completion, which leads to asynchronous hatching of the offspring. Hatching asynchrony creates an age and size hierarchy within a brood [5], [6]. During poor years, hatching asynchrony may serve as a mechanism to adjust brood size to food availability during the nestling period (“brood reduction hypothesis” [1]). It is typically the smallest/youngest siblings that die, those in which parents have invested the least in terms of time and energy [7].

Although hatching asynchrony is assumed to be the main factor influencing sibling competition and chick survival, mothers also influence the survival probability of individual chicks through differential allocation of egg mass, yolk hormones, yolk carotenoids and yolk antibodies [7]–[9]. Among the egg components, maternal yolk androgens have been proposed to play an important role in mediating sibling competition (see reviews in [8], [10], [11]). Maternal yolk androgens have been shown to influence the outcome of sibling competition through potential effects on embryonic developmental times, begging behaviour, post-hatching growth, and survival (see for example [12]–[14]).

Within-clutch variation in yolk androgen levels is therefore typically interpreted in the context of sibling competition. Decreasing levels of maternal yolk androgens with laying order are observed in some bird species exhibiting hatching asynchrony (for example, cattle egrets *Bubulcus ibis* [15], American coots *Fulica americana* [16] or zebra finches *Taeniopygia guttata* [17], [18]). They are thought to represent a mechanism for females to reinforce asymmetries between chicks, facilitating brood reduction [8]. On the other hand, increasing levels of maternal yolk androgens with laying order are found in other bird species also exhibiting hatching asynchrony (for example, canaries *Serinus canaria* [19], great tits *Parus major* [20] or black-headed gulls *Larus ridibundus* [21]). They have been interpreted as compensatory strategy by females for the competitive disadvantages suffered by later hatching chicks (“hatching asynchrony adjustment hypothesis” [8]).

Crested penguins (genus *Eudyptes*), including our study species, the southern rockhopper penguin *Eudyptes chrysocome*, have a unique pattern of hatching asynchrony in their two-egg clutches [22], [23]. The second-laid egg (B-egg) is 28% bigger and heavier than the first-laid egg (A-egg) (see [24]) and, although incubation starts only at clutch completion, the A-egg usually hatches one day after the B-egg [25]–[27]. Although both eggs commonly hatch, the chick hatching from the A-egg generally dies of starvation within a few days after hatching 27,28. The mechanisms by which reversed hatching asynchrony is achieved remain elusive [25]. Given the effects of yolk androgens on embryonic developmental times (see review in [29]), it has been suggested that differential allocation of yolk androgens may be an important mechanism to reverse the hatching pattern (see [30]). Indeed, in southern rockhopper penguins, B-eggs contain yolk androgen concentrations and total yolk androgen amounts at least 50% higher than A-eggs [30], . This was observed consistently for the three different androgens analysed (testosterone, androstenedione and dihydrotestosterone). Interestingly, clutches laid late in the breeding season had proportionally higher androgen levels in the B-egg compared to the A-egg than early clutches [30]. Late in the season, weather and food conditions may deteriorate and/or feeding parents may initiate pre-moult storage, and the likelihood that both chicks will be able to survive decreases (see [32]). Given the role that maternal yolk androgens play in mediating sibling competition (see above), we previously suggested that yolk androgens reinforce the competitive superiority of the chick hatched from B-egg (B-chick) when the survival of both chicks becomes unlikely [30].

In the present experimental study, we increased the levels of yolk androgens (testosterone, androstenedione and dihydrotestosterone) in freshly-laid eggs of free-living southern rockhopper penguins to test whether and to what extent yolk androgens contribute to the superiority of the B-chick. Given the dramatic differences in egg mass between A- and B-eggs [24], we excluded these potentially confounding effects by placing eggs of similar mass and the same position in the laying order into each foster nest. We examined the consequences of embryonic exposure to elevated yolk androgen levels on embryonic developmental times, chick growth and early survival, controlling for chick sex and the presence of a sibling during growth.

## Materials and Methods

### Ethical statement

The study was performed under proper legislation of the Belgian and Flemish law and was approved by the ethical committee on animal experimentation (ECD, ID numbers: 2011/44 and 2011/45). All work was conducted under a research license granted by the Environmental Planning Department of the Falkland Islands Government (Research Licence No: R06/2009). This license covered animal welfare in addition to the egg injection procedure. The methods that we used (nest check, egg manipulation, chick capture and measurement) probably created a low level of stress and did not cause any desertion from nestling activity or mortality. The impact of the injection itself is described in the manuscript. Manipulated clutches that failed to hatch any eggs were replaced with eggs found outside their own nest that we considered as lost by their original parents in order to avoid affecting the breeding success of the colony.

### General field procedures

The study was carried out at the “Settlement colony” (51°43′S, 61°17′W) on New Island, Falkland/Malvinas Islands from October to December 2010. In 2010, this colony held about 7500 breeding pairs of southern rockhopper penguins. Birds mainly breed in open rocky areas fringed by tussac grass *Poa flabellata*. The breeding biology at this large colony has been described previously [27]. Briefly, males arrive at the colony first (early October) and establish nest sites. Females arrive a few days later, for pairing and copulation in late October/early November. Laying and hatching intervals are relatively fixed; the second egg (B-egg) is generally laid four days after the first one (A-egg), incubation starts at clutch completion but the A-egg usually hatches one day after the B-egg (reversed hatching asynchrony).

During the laying period, we visited the study site daily, to mark and weigh freshly-laid eggs. From each of the monitored nests, which were homogeneously distributed within the study site, we randomly created one of two artificial nest categories (AA-nests with two A-eggs and BB-nests with two B-eggs). All egg pairs were matched for laying date (difference within foster clutches: mean ± SE [range]: 0.22±0.04 [0–1] day for AA-nests and no difference within foster clutches for BB-nests) and egg mass (difference within foster clutches: mean ± SE [range]: 4.68±0.27 [0–9.9] g for AA-nests and 1.67±0.18 [0–12.2] g for BB-nests) in order to obtain foster clutches with two foster-sibling eggs which were as similar as possible (for a similar design see [33]). We performed the yolk androgen injections on five consecutive days during the peak of the B-egg laying period. A-eggs were injected after clutch completion (four to six days after they were laid) and B-eggs were injected on the day after they were laid. The eggs were not incubated for longer than 24 h before androgen injection (normal incubation time: mean ± SD: 33.2±1.3 days for A-eggs and 32.1±1.0 days for B-eggs [27]). For each foster clutch, we injected one egg (control) with 50 µl sesame oil into the yolk while the second egg (androgen-treated) was injected with a mixture of androgens dissolved in 50 µl sesame oil. We injected a total of 400 eggs, creating 100 AA-nests and 100 BB-nests. All eggs were incubated in their foster nest after manipulation.

### Yolk androgen injection

The amount of each androgen injected into each A-egg was the amount needed to increase the total quantity of that androgen in a typical A-egg to the level found in a typical B-egg (see table 1). The amount of each androgen injected into each B-egg was such that the increase in concentration (relative to yolk mass) was the same as in an androgen-treated A-egg (see table 1). Data on yolk mass (mean 19.6 g in A-eggs and 22.5 g in B-eggs) and hormone levels were assessed in a number of previous studies [30], [31], [34], [35]. The injected amount of androgens corresponded on average to 2.61 times the standard deviation observed in natural clutches (table 1), which is comparable to previous injection studies [36]–[38].

**Table 1:** Amounts (in ng) of testosterone (T), androstenedione (A4) and dihydrotestosterone (DHT) injected into A- and B-eggs.

		Observed values					Injected amounts	
	Hormones	N	Min	Max	Mean	SD	in ng	in SD units
A-eggs	T	193	87	569	143.6	66.47	171	2.57
	A4	193	1140	9681	3361	1275	4154	3.26
	DHT	127	36	130	67.57	17.86	54	3.02
B-eggs	T	188	198	662	315.0	101.7	197	1.94
	A4	188	3006	14283	7516	1883	4782	2.54
	DHT	124	53	218	121.5	26.63	62	2.33

The amounts were calculated in relation to total yolk androgen amounts (minimum, maximum and mean ± Standard Deviation (SD), in ng) previously measured in A- and B-eggs of southern rockhopper penguins. Injected amounts are also shown as SD units, based on the SD observed in the non-manipulated population, for each androgen (T, A4 and DHT) and egg category (A- and B-eggs).

The same method was used to inject all eggs. Before injection, the eggs were left horizontal for a few minutes to allow the yolk to migrate towards the injection site (top of the equator). The injection site was carefully cleaned and disinfected with a pad impregnated with 70% isopropyl alcohol. A hole was drilled in the egg shell between the equator and the acute pole of the egg using a Dremel® Stylus™ Lithium-Ion with a sterile 0.9-mm bit. The solution was delivered into the yolk using a 1-ml syringe mounting 23-G sterile needle that was exchanged for each egg. The injection hole was then sealed with a piece of OpSite wound dressing (Smith & Nephew Medical Limited, Hull, England [13]) immediately after injection. Dissection of 10 eggs that were injected with a dye invariably revealed that the dye had been correctly injected into the yolk. After manipulation, eggs were placed under the incubating parents. All incubating parents stayed on the nests during the egg injection and after we returned the eggs.

### Egg and chick monitoring

From the beginning of the hatching period, we checked the foster nests twice a day (8:00 am and 8:00 pm) to keep track of the hatching pattern. We noted when the pipping process started (i.e. first crack in the egg shell) and when chicks were fully emerged. Two eggs started to hatch but were lost before we could classify them as fully hatched. Each new chick was weighed and measured within 12 h of hatching (hatching mass and size). The chicks were weighed to the nearest gram using a digital balance. We measured head length to the nearest 0.1 mm using a calliper and flipper length (extended from axilla) to the nearest millimetre with a ruler [39]. A drop of blood was taken from the brachial vein for molecular sexing. We were not able to obtain blood from one chick that died before its second capture. Newly hatched chicks were marked with a patch of non-toxic colour marker, later completed with a 23-mm glass encapsulated electronic transponder (TIRIS, Texas Instruments, USA) implanted under the skin of the upper back. After hatching, we checked foster nests daily for the survival and the identity of chicks during the first 13 days of early growth. During this period, chicks were also weighed and measured about every second day (interval: mean ± SE: 1.88±0.05 days). On a few occasions (i.e. when the weather conditions were too bad), we did not perform size measurements in order to minimise the chick manipulation time. All measurements were made by the same observer.

Eggs that failed to hatch (i.e. that were not hatched five days after their expected hatching date) were removed from the nests. If both eggs in a nest failed to hatch, we replaced them with one chick found outside its own nest that we considered as lost by its original parents. This guaranteed that all pairs could raise at least one chick, which is the typical brood size at fledging.

Sex determination was done with molecular techniques. About 1 µl of the blood sample was used for Chelex® resin-based DNA extraction [40]. Two µl of the resulting DNA solution was used in a polymerase chain reaction (PCR) to amplify a part of the CHD-W gene in females and the CHD-Z gene in both sexes (for details see [41]). The amplified products were separated in 1.5% agarose gels containing ethidium bromide. We evaluated the reliability with 10 individuals of known sex yielding a 100% correct match.

### Statistical analysis

Statistical analyses were conducted in SPSS 16.0 for Windows except for the Cox regression, which was conducted in Stata 12.1 for Windows. Differences in hatching success (hatched versus not hatched) and sex ratio between the two treatments were analysed using Generalized Estimating Equation (GEE) models with a logit link and binomial distribution. Foster nest identity was a repeated measure, treatments (control or androgen-treated) and nest category (AA- or BB-nest) were factors and egg mass (in g) was a covariate. We tested for the effects of elevated yolk androgen levels on embryonic developmental times and hatching measurements using GEE models for repeated measures (foster nest identity) with a linear link and Gaussian distribution, and with treatment, nest category and sex (male or female) as fixed factors and egg mass as a covariate. Nine hatched eggs, which were not incubated with a sibling egg until the end of the incubation, were removed from this analysis. Growth in body mass and size is approximately linear for southern rockhopper penguin chicks aged between 5 and 30 days (see [27]). Chick growth was therefore quantified using linear regressions of body mass, head length and flipper length according to chick age (in days) and using the growth rate (increase per day) for each individual with at least two measurements (see [42]). Because of the bad weather conditions, we were unable to calculate head and flipper growth rates for four chicks. We tested for the effects of elevated yolk androgen levels on chick growth using GEE models for repeated measures (foster nest identity) with a linear link and Gaussian distribution, and with treatment, nest category, sex and presence of a sibling chick in the nest during early growth (0 or 1) as fixed factors. Additional tests were performed running the same GEE model procedures for the chicks from control and androgen-treated eggs separately. We used Cox regression models to test for differences in early survival between chicks according to treatment, nest category, sex and presence of a sibling chick in the nest during early growth. Chick age was used as the time axis and robust standard errors, clustered by foster nest, were used to adjust for the non-independence of chicks raised in the same nest. Observations were right-censored at 13 days of age. In all the GEE and Cox regression procedures, we started with all the variables and their two-way interactions with treatment. We then simplified the models using a backward model selection procedure, starting with the least significant interaction. Main effects were not removed from the models, regardless of their significance. Values are presented as means ± standard errors (SE). Sample sizes are mentioned throughout the text.

## Results

Overall, out of the 200 eggs that were injected for each treatment, 72 control eggs and 47 androgen-treated eggs hatched. Twenty one broods were complete (12 AA-nests and 9 BB-nests) while 77 were incomplete (26 hatched only the androgen-treated egg and 51 hatched only the control egg). The hatching success of control eggs was higher than the hatching success of androgen-treated eggs (36% of the control eggs versus 23.5% of the androgen-treated eggs, *Wald χ^2^* = 8.21, *df* = 1, *P* = 0.004) while nest category and egg mass did not influence hatching success (*χ^2^* = 0.35, *df* = 1, *P* = 0.55 and *χ^2^* = 0.10, *df* = 1, *P* = 0.76, respectively). None of the interactions with treatment were significant (*χ^2^* = 0.32, *df* = 1, *P* = 0.57 for nest category and *χ^2^* = 1.30, *df* = 1, *P* = 0.25 for egg mass).

Out of the 116 eggs that hatched and were sexed (three chicks disappeared before blood sampling, see methods), 39 out of 70 chicks from control eggs and 22 out of 46 chicks from androgen-treated eggs were male. There was no difference in sex ratio between treatments (54% male for the control eggs versus 46% male for the androgen-treated eggs, *χ^2^* = 0.72, *df* = 1, *P* = 0.39). Nest category and egg mass also did not influence the sex ratio (*χ^2^* = 1.79, *df* = 1, *P* = 0.18 and *χ^2^* = 0.61, *df* = 1, *P* = 0.43, respectively).

### Embryonic developmental times and hatching measurements

The androgen treatment did not affect the embryonic developmental time to pipping (from the start of incubation to the first crack in the egg shell), the pipping duration (from the first crack in the egg shell to the fully emerged chick) or the total embryonic developmental time (sum of the two previous times; table 2). These three developmental times were not significantly different between the two nest categories or the two sexes (table 2).

**Table 2:** Test of the variation in embryonic developmental times and hatching measurements.

Dependent variable	Factors	B	*χ* ^2^	*P*
Developmental time to pipping	Treatment (androgen)	4.225	1.333	0.248
	Nest category (BB)	−8.884	1.360	0.244
	Sex (male)	−5.785	2.477	0.116
	Egg mass	0.125	0.204	0.651
Pipping duration	Treatment (androgen)	−0.632	0.110	0.740
	Nest category (BB)	−2.416	0.402	0.526
	Sex (male)	−1.115	0.331	0.565
	Egg mass	0.013	0.010	0.922
Total developmental time	Treatment (androgen)	3.593	0.993	0.319
	Nest category (BB)	−11.299	1.742	0.187
	Sex (male)	−6.900	2.948	0.086
	Egg mass	0.111	0.152	0.696
Hatching mass	Treatment (androgen)	1.000	1.052	0.305
	Nest category (BB)	1.400	0.565	0.452
	Sex (male)	0.196	0.037	0.847
	Egg mass	**0.737**	**102.412**	**<0.001**
Hatching head length	Treatment (androgen)	0.064	0.189	0.664
	Nest category (BB)	−0.128	0.186	0.666
	Sex (male)	0.203	1.960	0.161
	Egg mass	**0.068**	**41.845**	**<0.001**
Hatching flipper length	Treatment (androgen)	−0.098	0.346	0.557
	Nest category (BB)	−0.228	0.408	0.523
	Sex (male)	0.005	0.001	0.977
	Egg mass	**0.071**	**31.627**	**<0.001**

Results of the Generalized Estimating Equation (GEE) model procedures on embryonic developmental time to pipping (hours from the start of incubation to the first crack in the egg shell), pipping duration (hours from the first crack in the egg shell to the fully emerged chick), total embryonic developmental time (sum of the two previous times), hatching mass (g), hatching head length (mm) and hatching flipper length (mm) according to egg treatment (control or androgen-treated egg), nest category (AA- or BB-nest), sex (male or female) and egg mass (in g). All non-significant interactions were removed from the model during the backward procedure. *df* is always equal to 1. Significant *P*-values are marked in bold.

The androgen treatment did not significantly influence chicks' mass, head length or flipper length at hatching (table 2). There were no significant nest category and sex differences, but egg mass had a significant positive effect on all three of these hatchling measurements.

### Chick growth

**Figure 1:**
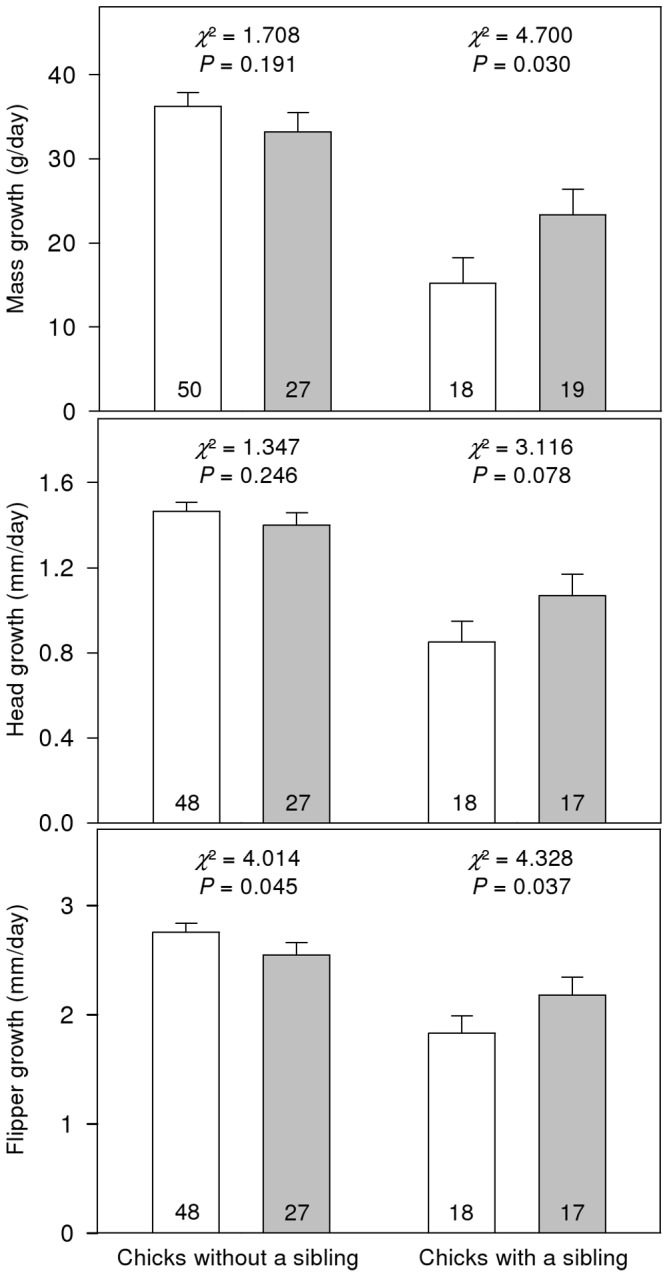
Growth rates of penguin chicks during the first 13 days after hatching. Differences in mass growth (in g/day), head growth (in mm/day) and flipper growth (in mm/day) are represented according to the presence of a sibling chick in the nest (left bars: chicks without a sibling; right bars: chicks with a sibling) and treatment (white bars: chicks from control eggs; grey bars: chicks from androgen-treated eggs). Bars show means ± Standard Errors. The significance of the difference between treatments obtained from Generalized Estimating Equation (GEE) procedures with treatment (control or androgen-treated egg), nest category (AA- or BB-nest) and sex (male or female) as factors and foster nest identity as a repeated measure are presented above respective bars within each sibling presence category. The sample size is given at the base of each bar.

The androgen treatment significantly influenced chick growth in interaction with the presence of a sibling chick in the nest (table 3). Chicks from androgen-treated eggs had significantly faster mass and flipper growth rates than chicks from control eggs when they had a sibling (fig. 1). This difference was also almost significant for head growth rate. In other words, chicks from control eggs had a much slower growth rates when they did have a sibling than when they did not (mass growth: *χ* ^2^ = 42.03, *df* = 1, *P*<0.001; head growth: *χ* ^2^ = 34.68, *df* = 1, *P*<0.001; flipper growth: *χ* ^2^ = 28.76, *df* = 1, *P*<0.001) while this difference according to the presence of a sibling was not consistently significant for chicks from androgen-treated eggs (mass growth: *χ* ^2^ = 1.73, *df* = 1, *P* = 0.19; head growth: *χ* ^2^ = 4.17, *df* = 1, *P* = 0.04; flipper growth: *χ* ^2^ = 0.79, *df* = 1, *P* = 0.37). In addition, head growth and flipper growth were significantly faster for males than for females (head growth: 1.39±0.05 mm/day, *n* = 57 for males and 1.18±0.05 mm/day, *n* = 53 for females; flipper growth: 2.62±0.09 mm/day, *n* = 57 for males and 2.30±0.10 mm/day, *n* = 53 for females; table 3).

**Table 3:** Test of the variation in mass and size growths.

Dependent variable	Factors	B	*χ* ^2^	*P*
Mass growth	Treatment (androgen)	−3.644	1.778	0.182
	Nest category (BB)	3.686	3.184	0.074
	Sex (male)	4.025	3.055	0.081
	Sibling presence (1)	−**20.867**	**41.980**	**<0.001**
	Treatment×Sibling presence	**14.208**	**6.001**	**0.014**
Head growth	Treatment (androgen)	−0.081	1.338	0.247
	Nest category (BB)	0.023	0.182	0.670
	Sex (male)	**0.163**	**6.547**	**0.011**
	Sibling presence (1)	−**0.608**	**34.292**	**<0.001**
	Treatment×Sibling presence	**0.346**	**4.369**	**0.037**
Flipper growth	Treatment (androgen)	**−0.264**	**3.945**	**0.047**
	Nest category (BB)	0.216	3.745	0.053
	Sex (male)	**0.303**	**6.844**	**0.009**
	Sibling presence (1)	−**0.914**	**28.659**	**<0.001**
	Treatment×Sibling presence	**0.730**	**8.218**	**0.004**

Results of the Generalized Estimating Equation (GEE) model procedures on mass growth (g/day), head growth (mm/day) and flipper growth (mm/day) according to egg treatment (control or androgen-treated egg), nest category (AA- or BB-nest), sex (male or female) and the presence of a sibling chick in the nest during early growth (0 or 1). All non-significant interactions were removed from the model during the backward procedure. *df* is always equal to 1. Significant *P*-values are marked in bold.

### Early survival

**Figure 2:**
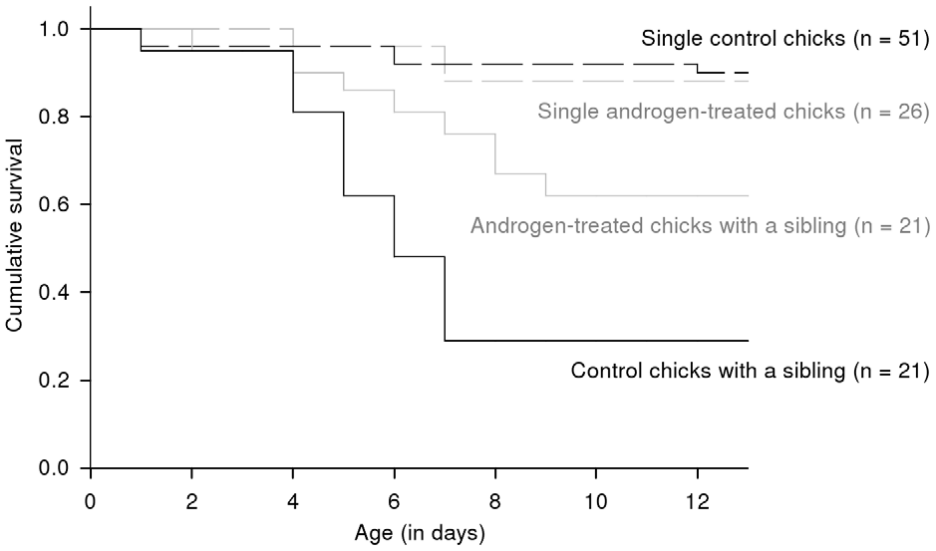
Early survival of the injected southern rockhopper penguin chicks during the first 13 days after hatching. Differences are shown according to egg treatment (black lines: control eggs; grey lines: androgen-treated eggs) and the presence of a sibling chick in the nest (dashed lines: single chick without a sibling; solid lines: chick with a sibling). The sample size is given between brackets for each of these four groups.

The presence of a sibling significantly reduced the survival probability of a chick (table 4). However, there was also some evidence suggesting that early survival depended on androgen treatment in the presence of a sibling, but not when a chick was alone. The interaction between treatment and sibling presence was the last to be removed from the model (mortality hazard ratio = 0.23, *z* = −1.61, *P* = 0.11). We nevertheless investigated this further by conducting separate tests (as performed on chick growth, and including all main effects) on chicks with and without a sibling. In the absence of a sibling, treatment did not significantly influence early survival (mortality hazard ratio = 1.53, *z* = 0.57, *P* = 0.57; fig. 2). However, in the presence of a sibling the androgen treatment significantly increased early survival (mortality hazard ratio = 0.35, *z* = −2.19, *P* = 0.03; fig. 2). Among the 21 treatment-control pairs of chicks in which both hatched successfully, there were 11 in which only the androgen-treated chick survived to 13 days and 4 in which only the control chick did so (there were 2 nests in which both survived, and 4 nests where both chicks died). Nest category and sex did not significantly influence chick early survival (table 4).

**Table 4:** Hazard ratios for early chick mortality according to egg treatment (control or androgen-treated egg), nest category (AA- or BB-nest), sex (male or female) and the presence of a sibling chick in the nest during early growth (0 or 1).

Factors	Hazard ratio	*z*	*P*	*[95% conf. Interval]*	
Treatment (androgen)	0.501	−1.63	0.103	0.218	1.151
Nest category (BB)	0.620	−1.24	0.214	0.291	1.318
Sex (male)	1.045	0.14	0.885	0.571	1.913
Sibling presence (1)	**8.509**	**4.81**	**<0.001**	**3.554**	**20.370**

Cox proportional hazards models were used. All non-significant interactions were removed from the model during the backward procedure. *df* is always equal to 1. Significant *P*-values are marked in bold.

## Discussion

By increasing the amount of yolk androgens in freshly-laid eggs of southern rockhopper penguins, a species with reversed hatching asynchrony, we investigated whether yolk androgens contribute to the superiority of the B-chick, through changes in developmental time or growth. While the androgen treatment did not influence embryonic developmental time and chick measurements at hatching, it indeed affected chick growth in a context-dependent manner and improved chick early survival in two-chick broods.

### Embryonic development

In order to explain the shorter embryonic developmental times for B-eggs compared to A-eggs, we proposed that high yolk androgen levels, as observed in B-eggs, may play a significant role in reversing the hatching pattern in southern rockhopper penguins [30]. However, we did not find evidence for shorter embryonic developmental times for androgen-treated eggs compared to control eggs. This was the case for the three different developmental time measures we looked at. We are, therefore, unable to validate our previous hypothesis. However, while the effects of androgen treatment on embryonic developmental times appear to be consistent within species (for example in black-headed gulls [13], [43] and zebra finches [14], [44]), they have been observed to be inconsistent across species. Previous studies have reported either shortened times to hatching [13], [33], 43,45, postponement of hatching [14], [44], [46] or no influence on embryonic developmental times (see for example [47]–[49]). The reasons for these differences between species are still elusive, and it thus remains unclear why yolk androgens could not be shown to affect embryonic development in our study species. However, part of the between-species variation could be due to differences in the type of hormones injected: some studies manipulating several androgens, while others manipulated only a single androgen (e.g. only testosterone for yellow-legged gull *Larus michahellis* [47], [49] but testosterone and androstenedione for black-headed gull [13], [43]).

Interestingly, we did not find differences in developmental times between A- and B-eggs, as have been reported previously under natural conditions (see for examples [27], [28]). The eggs here were incubated with a sibling egg of similar mass. This, in combination with a previous study showing no differences in developmental time in single incubated eggs [27] strongly suggests that the egg size asymmetry between A- and B-eggs drives the reversal of the hatching pattern. The larger B-egg may get a better incubation position and closer contact with the brood patch (see [50] for a discussion on this subject) facilitating embryonic development.

When interpreting the results, it has to be taken into account that the hatching failure was rather high (70%, but see [13], [14], [51], [52] for similar negative effects on hatching successes with 61–69% hatching failure). This may relate to a high natural rate of hatching failure that we previously observed for the entirely untreated eggs of this population (34%, [27]). Yet, the hatching success also differed between treatments. The mechanism(s) and cause(s) behind this bias remain unclear, but testosterone has been shown to cause developmental arrest in embryos of other animal species (see [53]). The androgen levels reached after injection (an average increase of 2.61 standard deviations) could also have contributed to the hatching failures, as the elevation is somewhat higher than the 2 standard deviations typically injected [36]–[38]. The observed negative effects on hatchability may, independently of the mechanism(s) involved, form a cost of maternal androgen deposition that has to be considered when discussing the costs and benefits of yolk androgens. It may give rise to a biased sample in the androgen-treated group compared to the control group, although the treatment groups did not differ in the mass, size and sex of chicks at hatching, which argues against the idea that the treatment did select for the high quality offspring.

### Chick growth and survival

The effect of treatment on chick growth and survival differed according to the presence or absence of a sibling, pointing towards nutritional limitations in the context of sibling competition. The androgen treatment benefitted chick growth in interaction with the number of siblings in a brood. In the presence of a sibling, chicks from androgen-treated eggs had faster growth rates than chicks from control eggs. Under these circumstances they also had a higher survival probability. In other words, chicks from control eggs suffered more from sibling competition, in terms of reduced growth and survival, while chicks from androgen-treated eggs suffered much less from sibling competition. Although males grew more quickly than females, as previously reported in this species [39], we did not find a sex difference in the response to the androgen treatment (see [44], [54], [55], but see [49]).

As suggested by Eising et al. [13], the effect of yolk androgens on chick growth and survival could be at least partly mediated by their effect on sibling competition. Similarly to plasma androgens (see [56], [57] for reviews), yolk androgens could increase aggressive behavioural traits [43] and begging for parental food [33], providing a competitive advantage for B-chicks (see reviews in [8], [10], [11]). Yolk androgens may also directly accelerate growth and development [11], which in turn allows better access to food resources (from feeding females) if sibling competition is size dependent. Alternatively, parental favouritism in food allocation could have been indirectly affected by the androgen treatment through an effect on plumage or beak colouration for example (but see [12]). These three processes may have promoted the early survival pattern observed in this study (see also [44], [58]). Future studies including behavioural observations would be necessary to disentangle the effects of each of these potential mechanisms.

In conclusion, we did not find any evidence that yolk androgens contribute to the reversed hatching pattern in southern rockhopper penguins. After hatching, however, we show that elevated yolk androgen levels benefit the chicks in terms of their early growth and to some extent survival in the context of sibling competition. This is in line with the idea that maternal yolk androgens adjust offspring phenotype to specific environmental conditions, implying that the costs and benefits of yolk androgens depend on the environmental circumstances. These benefits may indeed have to be traded-off against the negative effects of elevated yolk androgens such as on hatchability. Higher yolk androgen levels in B-eggs could be an adaptive strategy for females to further enhance the superiority of the (mostly older and larger) B-chick, enabling a quicker elimination of A-chicks under unfavourable conditions. This may also explain why late clutches had proportionally higher androgen levels in the B-egg than early clutches [30].

## References

[1] Lack D (1954) The natural regulation of animal numbers. Oxford: Clarendon.

[2] Mock DW, Parker GA (1998) Siblicide, family conflict and the evolutionary limits of selfishness. Animal Behaviour 56: 1–10. 9710456 10.1006/anbe.1998.0842

[3] Mock DW, Parker GA(1997) The evolution of sibling rivalry. Oxford University Press.

[4] Lack D (1947) The significance of clutch-size. Ibis 89: 302–352.

[5] Amundsen T, Slagsvold T (1991) Hatching asynchrony: facilitating adaptive or maladaptive brood reduction? In: Acta XX Congressus Internationalis Ornithologici. Bell BD, Cossee RO, Flux JEC, Heather BD, et al, editors. Wellington, New Zealand 1707–1719.

[6] Magrath RD (1990) Hatching asynchrony in altricial birds. Biological Reviews 65: 587–622.

[7] Howe HF (1978) Initial investment, clutch size, and brood reduction in the common grackle (*Quiscalus quiscula* L.). Ecology 59: 1109–1122.

[8] Groothuis TGG, Müller W, von Engelhardt N, Carere C, Eising C (2005) Maternal hormones as a tool to adjust offspring phenotype in avian species. Neuroscience and Biobehavioral Reviews 29: 329–352. 15811503 10.1016/j.neubiorev.2004.12.002

[9] Saino N, Bertacche V, Ferrari RP, Martinelli R, Møller AP, et al (2002) Carotenoid concentration in barn swallow eggs is influenced by laying order, maternal infection and paternal ornamentation. Proceedings of the Royal Society of London, Series B: Biological Sciences 269: 1729–1733. 12204135 10.1098/rspb.2002.2088 PMC1691081

[10] Gil D (2008) Hormones in avian eggs: physiology, ecology and behavior. Advances in the Study of Behavior 38: 337–398.

[11] Groothuis TGG, Schwabl H (2008) Hormone-mediated maternal effects in birds: mechanisms matter but what do we know of them? Philosophical Transactions of the Royal Society of London, series B: Biological Sciences 363: 1647–1661. 18048291 10.1098/rstb.2007.0007 PMC2606725

[12] Müller W, Boonen S, Groothuis TGG, Eens M (2010) Maternal yolk testosterone in canary eggs: toward a better understanding of mechanisms and function. Behavioral Ecology 21: 493–500.

[13] Eising CM, Eikenaar C, Schwabl H, Groothuis TGG (2001) Maternal androgens in black-headed gull (*Larus ridibundus*) eggs: consequences for chick development. Proceedings of the Royal Society of London, Series B: Biological Sciences 268: 839–846. 11345330 10.1098/rspb.2001.1594 PMC1088678

[14] Boncoraglio G, Groothuis TGG, von Engelhardt N (2011) Differential maternal testosterone allocation among siblings benefits both mother and offspring in the zebra finch *Taeniopygia guttata* . American Naturalist 178: 64–74. 10.1086/660278 21670578

[15] Schwabl H, Mock DW, Gieg JA (1997) A hormonal mechanism for parental favouritism. Nature 386: 231.9069278

[16] Reed WL, Vleck CM (2001) Functional significance of variation in egg-yolk androgens in the American coot. Oecologia 128: 164–171. 28547464 10.1007/s004420100642

[17] Gil D, Heim C, Bulmer E, Rocha M, Puerta M, et al (2004) Negative effects of early developmental stress on yolk testosterone levels in a passerine bird. Journal of Experimental Biology 207: 2215–2220. 15159426 10.1242/jeb.01013

[18] Gil D, Graves J, Hazon N, Wells A (1999) Male attractiveness and differential testosterone investment in zebra finch eggs. Science 286: 126–128. 10506561 10.1126/science.286.5437.126

[19] Gil D, Leboucher G, Lacroix A, Cue R, Kreutzer M (2004) Female canaries produce eggs with greater amounts of testosterone when exposed to preferred male song. Hormones and Behavior 45: 64–70. 14733893 10.1016/j.yhbeh.2003.08.005

[20] Tschirren B, Richner H, Schwabl H (2004) Ectoparasite-modulated deposition of maternal androgens in great tit eggs. Proceedings of the Royal Society of London, Series B: Biological Sciences 271: 1371–1375. 15306335 10.1098/rspb.2004.2730 PMC1691743

[21] Groothuis TG, Schwabl H (2002) Determinants of within- and among-clutch variation in levels of maternal hormones in black-headed gull eggs. Functional Ecology 16: 281–289.

[22] Warham J (1975) The crested penguins. In: The biology of penguins. Stonehouse B, editor. London, UK: The Macmillan Press 189–269.

[23] Williams TD(1995) The penguins. Oxford, UK: Oxford University Press.

[24] Demongin L, Poisbleau M, Raya Rey A, Schiavini A, Quillfeldt P, et al (2010) Geographical variation in egg size dimorphism in rockhopper penguins. Polar Biology 33: 469–476.

[25] St Clair CC (1996) Multiple mechanisms of reversed hatching asynchrony in rockhopper penguins. Journal of Animal Ecology 65: 485–494.

[26] St Clair CC (1998) What is the function of first eggs in crested penguins? Auk 115: 478–482.

[27] Poisbleau M, Demongin L, Strange IJ, Otley H, Quillfeldt P (2008) Aspects of the breeding biology of the southern rockhopper penguin *Eudyptes c. chrysocome* and new consideration on the intrinsic capacity of the A-egg. Polar Biology 31: 925–932.

[28] Lamey TC (1990) Hatch asynchrony and brood reduction in penguins. In: Penguin biology. Davis LS, Darby JT, editors. San Diego: Academic Press 399–416.

[29] von Engelhardt N, Groothuis TGG (2011) Maternal hormones in avian eggs. In: Hormones and reproduction of vertebrates. Norris DO, Lopez KH, editors. Academic Press 91–127.

[30] Poisbleau M, Demongin L, Chastel O, Eens M, Quillfeldt P (2011) Yolk androgen deposition in rockhopper penguins, a species with reversed hatching asynchrony. General and Comparative Endocrinology 170: 622–628. 21130090 10.1016/j.ygcen.2010.11.027

[31] Poisbleau M, Carslake D, Demongin L, Eens M, Chastel O, et al (2011) Yolk androgen deposition without an energetic cost for female rockhopper penguins: a compensatory strategy to accelerate brood reduction? Biology Letters 7: 605–607. 21325311 10.1098/rsbl.2010.1134 PMC3130214

[32] Moreno J, Barbosa A, Potti J, Merino S (1997) The effects of hatching date and parental quality on chick growth and creching age in the chinstrap penguin (*Pygoscelis antarctica*): a field experiment. Auk 114: 47–54.

[33] Eising CM, Groothuis TGG (2003) Yolk androgens and begging behaviour in black-headed gull chicks: an experimental field study. Animal Behaviour 66: 1027–1034.

[34] Poisbleau M, Demongin L, Angelier F, Dano S, Lacroix A, et al (2009) What ecological factors can affect albumen corticosterone levels in the clutches of seabirds? Timing of breeding, disturbance and laying order in rockhopper penguins (*Eudyptes chrysocome chrysocome*). General and Comparative Endocrinology 162: 139–145. 19341737 10.1016/j.ygcen.2009.03.022

[35] Poisbleau M, Demongin L, Trouve C, Quillfeldt P (2009) Maternal deposition of yolk corticosterone in clutches of southern rockhopper penguins (*Eudyptes chrysocome chrysocome*). Hormones and Behavior 55: 500–506. 19232349 10.1016/j.yhbeh.2009.02.002

[36] Barnett CA, Clairardin SG, Thompson CF, Sakaluk SK (2011) Turning a deaf ear: a test of the manipulating androgens hypothesis in house wrens. Animal Behaviour 81: 113–120.

[37] Romano M, Rubolini D, Martinelli R, Alquati AB, Saino N (2005) Experimental manipulation of yolk testosterone affects digit length ratios in the ring-necked pheasant (*Phasianus colchicus*). Hormones and Behavior 48: 342–346. 15878573 10.1016/j.yhbeh.2005.03.007

[38] Bonisoli-Alquati A, Matteo A, Ambrosini R, Rubolini D, Romano M, et al (2011) Effects of egg testosterone on female mate choice and male sexual behavior in the pheasant. Hormones and Behavior 59: 75–82. 21029735 10.1016/j.yhbeh.2010.10.013

[39] Poisbleau M, Demongin L, van Noordwijk HJ, Strange IJ, Quillfeldt P (2010) Sexual dimorphism and use of morphological measurements to sex adults, immatures and chicks of rockhopper penguins. Ardea 98: 217–224.

[40] Walsh PS, Metzger DA, Higuchi R (1991) Chelex-100 as a medium for simple extraction of DNA for PCR-based typing from forensic material. Biotechniques 10: 506–513. 1867860

[41] Griffiths R, Double MC, Orr K, Dawson RJG (1998) A DNA test to sex most birds. Molecular Ecology 7: 1701–1075. 10.1046/j.1365-294x.1998.00389.x 9711866

[42] Hinde CA, Buchanan KL, Kilner RM (2009) Prenatal environmental effects match offspring begging to parental provisioning. Proceedings of the Royal Society of London, Series B: Biological Sciences 276: 2787–2794. 19419982 10.1098/rspb.2009.0375 PMC2839951

[43] Müller W, Dijkstra C, Groothuis TGG (2009) Maternal yolk androgens stimulate territorial behaviour in black-headed gull chicks. Biology Letters 5: 586–588. 19515649 10.1098/rsbl.2009.0283 PMC2781951

[44] von Engelhardt N, Carere C, Dijkstra C, Groothuis TGG (2006) Sex-specific effects of yolk testosterone on survival, begging and growth of zebra finches. Proceedings of the Royal Society of London, Series B: Biological Sciences 273: 65–70. 16519236 10.1098/rspb.2005.3274 PMC1560008

[45] Müller W, Eens M (2009) Elevated yolk androgen levels and the expression of multiple sexually selected male characters. Hormones and Behavior 55: 175–181. 18976657 10.1016/j.yhbeh.2008.09.012

[46] Sockman KW, Schwabl H (2000) Yolk androgens reduce offspring survival. Proceedings of the Royal Society of London, Series B: Biological Sciences 267: 1451–1456. 10983830 10.1098/rspb.2000.1163 PMC1690699

[47] Boncoraglio G, Rubolini D, Romano M, Martinelli R, Saino N (2006) Effects of elevated yolk androgens on perinatal begging behavior in yellow-legged gull (*Larus michahellis*) chicks. Hormones and Behavior 50: 442–447. 16842788 10.1016/j.yhbeh.2006.05.005

[48] Andersson S, Uller T, Lõhmus M, Sundström F (2004) Effects of egg yolk testosterone on growth and immunity in a precocial bird. Journal of Evolutionary Biology 17: 501–505. 15149393 10.1111/j.1420-9101.2004.00706.x

[49] Rubolini D, Romano M, Martinelli R, Saino N (2006) Effects of elevated yolk testosterone levels on survival, growth and immunity of male and female yellow-legged gull chicks. Behavioral Ecology and Sociobiology 59: 344–352.

[50] Massaro M, Davis LS (2004) Preferential incubation positions for different sized eggs and their influence on incubation period and hatching asynchrony in Snares crested (*Eudyptes robustus*) and yellow-eyed penguins (*Megadyptes antipodes*). Behavioral Ecology and Sociobiology 56: 426–434.

[51] Daisley JN, Bromundt V, Möstl E, Kotrschal K (2005) Enhanced yolk testosterone influences behavioral phenotype independent of sex in Japonese quail chicks *Coturnix japonica* . Hormones and Behavior 47: 185–194. 15664022 10.1016/j.yhbeh.2004.09.006

[52] Navara KJ, Hill GE, Mendonça MT (2005) Variable effects of yolk androgens on growth, survival, and immunity in eastern bluebird nestlings. Physiological and Biochemical Zoology 78: 570–578. 15957111 10.1086/430689

[53] Mu X, LeBlanc GA (2002) Developmental toxicity of testosterone in the crustacean *Daphnia magna* involves anti-ecdysteroidal activity. General and Comparative Endocrinology 129: 127–133. 12441123 10.1016/s0016-6480(02)00518-x

[54] Rutkowska J, Wilk T, Cichoń M (2007) Androgen-dependent maternal effects on offspring fitness in zebra finches. Behavioral Ecology and Sociobiology 61: 1211–1217.

[55] Sockman KW, Weiss J, Webster MS, Talbott V, Schwabl H (2008) Sex-specific effects of yolk-androgens on growth of nestling American kestrels. Behavioral Ecology and Sociobiology 62: 617–625.

[56] Ketterson ED, Nolan V Jr (1992) Hormones and life histories: an integrative approach. American Naturalist 140: S33–S62. 10.1086/285396 19426026

[57] Wingfield JC, Lynn SE, Soma KK (2001) Avoiding the ‘costs’ of testosterone: ecological bases of hormone-behavior interactions. Brain, Behavior and Evolution 57: 239–251. 10.1159/000047243 11641561

[58] Müller W, Vergauwen J, Eens M (2009) Long-lasting consequences of elevated yolk testosterone levels on female reproduction. Behavioral Ecology and Sociobiology 63: 809–816.

